# Safety and adverse events following COVID‐19 vaccination among people with epilepsy: A cross‐sectional study

**DOI:** 10.1002/epi4.12658

**Published:** 2022-11-21

**Authors:** Marjorie Jia Yi Ong, Ching Soong Khoo, Yi Xuan Lee, Vaanee Poongkuntran, Chia Khoi Tang, Yu Joe Choong, Rozita Hod, Hui Jan Tan

**Affiliations:** ^1^ Neurology Unit, Department of Medicine Universiti Kebangsaan Malaysia Medical Centre Kuala Lumpur Malaysia; ^2^ Department of Community Health Universiti Kebangsaan Malaysia Medical Centre Kuala Lumpur Malaysia

**Keywords:** adverse events following immunization, COVID‐19 vaccination, epilepsy

## Abstract

**Objective:**

Epilepsy is a non‐communicable disease costing a massive burden globally. It is known that there is increased prevalence of morbidity and mortality following COVID‐19 infection among people with epilepsy (PWE). However, there is limited information about the adverse events following COVID‐19 immunization among PWE. Hence, this study aimed to assess the safety and adverse events following immunization (AEFI) of various COVID‐19 vaccines among PWE from our centre, focusing on neurological AEFI.

**Methods:**

This cross‐sectional study recruited 120 adult PWE from the Neurology Clinic of the Universiti Kebangsaan Malaysia Medical Centre (UKMMC). Consent‐taking was conducted via synchronous or asynchronous approaches, followed by a phone call interview session. The interview collected socio‐demographic information, epilepsy‐related variables, and vaccination‐related variables. Univariate analysis and multiple logistic regression analysis were done to confirm factors associated with the AEFI of COVID‐19 vaccination.

**Results:**

Among all types of COVID‐19 vaccines, most of the PWE received the Cominarty® COVID‐19 vaccination (52.5%). Overall, local AEFI was the quickest to develop, with an average onset within a day. PWE with normal body mass index (BMI) had a higher risk of developing both local and systemic AEFI compared to those underweight and obese PWE (OR: 15.09, 95% CI 1.70–134.28, *P* = 0.02).

**Significance:**

COVID‐19 vaccines are safe for PWE. AEFI among PWE are similar to those of the general population following COVID‐19 vaccination. Therefore, clinicians should encourage PWE to take COVID‐19 vaccines.

**Key Points**

- The COVID‐19 vaccines are safe and well tolerated among people with epilepsy (PWE).
- The adverse events following immunization (AEFI) among PWE are similar to those of the general population.
- Normal body mass index (BMI) is associated with a higher risk of developing AEFI.
- One of the most serious neurological AEFI of COVID‐19 vaccines reported among PWE is the development of new seizures requiring hospitalization.

## 1 INTRODUCTION

### 1.1 Background

Epilepsy is a non‐communicable disease contributing to an inevitable local and global burden on public health. More than 50 million people worldwide are identified with epilepsy, costing more than 13 million disability‐adjusted life years (DALYs) globally. The lives of people with epilepsy (PWE) are often impacted by negativity, such as stigma and discrimination.^1^ It is reported by Cabezudo‐Garc ´ıa and colleagues that active epilepsy was associated with increased prevalence of severity and fatality of COVID‐19 infection.^2^, ^3^, ^4^ The more comorbidities one has, the greater is the risk of more severe COVID‐19 infection.^2^, ^3^, ^5^ Providentially, vaccinations against the coronavirus were successfully developed, massively produced, and administered globally. In spite of that, the concerns of the adverse events following immunization (AEFI) among PWE have been unequivocal.

Vaccines that were approved by the Food and Drug Administration (FDA) for emergency use in the United States against COVID‐19 include the Pfizer‐BioNtech vaccine, followed by AstraZeneca, and Johnson and Johnson. The preliminary evidence from the safety and efficacy trials of these vaccines has been favorable.^6^, ^7^, ^8^ According to the International League Against Epilepsy (ILAE), PWE are encouraged to receive the COVID‐19 vaccine as the benefits outweigh the risks, namely the impact of COVID‐19 infection and its complications are more severe than the AEFI. The seizures developed are more likely due to fever following COVID‐19 vaccination, the risks of which can be reduced by taking antipyretics regularly for 48 hours after the vaccination. According to the ILAE, there is currently no evidence proving that having epilepsy has a strong relation to the increased risk of AEFI from any COVID‐19 vaccines.^9^ As a matter of fact, as per the Clinical Guidelines on COVID‐19 Vaccination in Malaysia, PWE are classified under vaccine priority groups as the underlying medical condition may increase the risk of severe illness from COVID‐19. CanSinoBio vaccine is by far the only COVID‐19 vaccine that has clear contraindications for people with uncontrolled epilepsy and other progressive neurological diseases, which is not available in Malaysia. In Malaysia, the three approved COVID‐19 vaccines are the Pfizer‐BioNtech, AstraZeneca and Sinovac.^10^

As COVID‐19 variants with the potential to compromise vaccine efficacy continue to develop globally, additional data on the real‐world safety and efficacy of our present mass immunization initiatives is required especially in patients with chronic disorders like PWE. The real‐world data on its efficacy focusing on certain priority groups are still relatively low given the period the mass vaccination programme began. Therefore, we conducted a study to assess the safety and AEFI of COVID‐19 vaccination among PWE focusing on neurological AEFI in relation to the socio‐demographic, epilepsy, and vaccination factors. We aimed to share a baseline reference with our data as a novel study in Malaysia.

### 1.2 Operational definition

According to the ILAE, epileptic seizure is defined as a transient occurrence of signs and/or symptoms due to abnormal excessive or synchronous neuronal activity in the brain. Epilepsy is a chronic neurological disorder characterized by an enduring predisposition to generate epileptic seizures. The diagnosis of epilepsy usually requires the occurrence of at least two seizures more than 24 hours apart.^11^

Based on the World Health Organization (WHO) Global Manual on Surveillance of Adverse Events (2014), an adverse event following immunization (AEFI) is any untoward medical occurrence which follows immunization and does not necessarily have a causal relationship with the usage of the vaccine. The adverse event may be any unfavorable or unintended sign, abnormal laboratory finding, symptom or disease. Reported adverse events can either be true adverse events i.e., resulting from the vaccine or immunization process, or coincidental events that are not due to the vaccine or immunization process but are temporarily associated with immunization. A serious AEFI is an AEFI which results in death, is life‐threatening, requires in‐patient hospitalization or prolongation of existing hospitalization, results in persistent or significant disability or incapacity, is a congenital anomaly or birth defect, or requires intervention to prevent permanent impairment or damage.^12^

According to a nationwide descriptive study in Mexico by García‐Grimshaw and colleagues in 2021, neurologic AEFI was defined as any sign or symptom of AEFI potentially related to central or peripheral nervous system dysfunction, which includes headache, motor symptoms, sensory symptoms (paraesthesia, dysesthesia, numbness, pinprick, tingling, or a combination thereof), focalising signs, altered mental status (including syncope), and weakness (paresis, weakness, diminished strength, lack of strength, paralysis, or a combination thereof). Headache, sensory symptoms, weakness, and syncope were considered non‐serious neurologic adverse events unless otherwise defined by the medical personnel or if fulfilling the definition of serious AEFI.^13^

## 2 METHODS

### 2.1 Sampling

This was a cross‐sectional study conducted at the Neurology Clinic, Universiti Kebangsaan Malaysia Medical Centre (UKMMC), which is a tertiary hospital in Malaysia. Around 500 PWE are regularly followed up at our hospital. The sample size for this study was determined using a two‐tailed z‐test of proportions between two groups with 80% power and a 5% level of significance. In reference to Massoud and colleagues, the prevalence of vaccination adverse effects among PWE on single anti‐seizure medication (ASM) therapy was 36.4%, with an attrition rate of 20% for wrong entries or poor mobile signals. For our study, 120 samples were required.^14^

### 2.2 Data collection

Patients with epilepsy coming to our clinic between November 8, 2021 and January 12, 2022 were invited to participate in the study. We only recruited PWE, who fulfilled the following criteria: Malaysian nationality, aged at least 18 years old in 2021, having a definite diagnosis of epilepsy according to the ILAE Guidelines, able to communicate in Malay/English/Mandarin/Tamil, having completed at least two doses of COVID‐19 vaccination. The first 120 PWE fulfilling the criteria were invited to join the study via asynchronous hardcopy consent‐taking during their consultation session at the clinic or synchronously over the phone consent‐taking, both of which were followed by a phone call interview session. The interviews were held based on a scripted measure, formatted on Microsoft Forms and only accessible by the authors. All over‐the‐phone consent‐taking and interviews were recorded. For those who were unable to partake in the interview due to speech limitations including mutism and presence of speech disturbance, impaired cognitive status, fatigue, and weakness, or in a comatose state, the caregiver or next‐of‐kin was allowed to proceed with the consent‐taking and interview on behalf of the PWE.

### 2.3 Instrument

A scripted and standardized online measure was developed on the Microsoft Forms portal. The measure consisted of three sections: socio‐demographic information, epilepsy‐related variables, and vaccination‐related variables. Socio‐demographic information of the PWE included gender, race, age, body mass index (BMI) in accordance with the Malaysian Clinical Practice Guideline on Management of Obesity 2004 (BMI <18.5 kg/m^2^ is underweight; 18.5–22.9 kg/m^2^ is normal; ≤23 kg/m^2^ is overweight; 23.0–27.4 kg/m^2^ is overweight or pre‐obese; 27.5–34.9 kg/m^2^ is Obese I; 35.0–39.9 kg/m^2^ is Obese II; ≥40 kg/m^2^ is Obese III), education level in accordance with the education system of the Malaysian Ministry of Education and Higher Education (Pre‐school education: education programme for students aged 4‐6; Primary education: 6 years of primary level course of study; Secondary education: consists of lower and upper secondary education; Pre‐university: Form 6, matriculation, International Baccalaureate or other equivalent; Undergraduate: bachelor degree levels; Postgraduate: master degrees and PhD levels), marital status, employment status, allergy history, other medical illnesses (congenital diseases, metabolic syndromes, mental disorders), history of surgery, and alternative medications (conventional medicine that is not fully integrated into the dominant health‐care system in Malaysia; also includes traditional medicine).^15^, ^16^, ^17^

Epilepsy‐related variables included the age of first onset of seizure, age of definite diagnosis of epilepsy, type of epilepsy, pre‐vaccination seizure frequency, difference in seizure frequency between pre‐vaccination and post**‐**vaccination, history of status epilepticus within 3 months before the first dose of vaccination, number, and type of current antiseizure medication (ASM), and medication adherence. Patients' latest diagnosis and electroencephalographic findings were collaboratively assessed and classified according to the epilepsy types stated in the ILAE Classification of the Epilepsies 2017.^18^ The subjects were interviewed about their monthly, 6‐monthly, and yearly frequency of seizures before the first dose of vaccination. The frequency was accessed and further classified into seizure‐free, lower seizure frequency (≤10 seizures per year), and higher seizure frequency (>10 seizures per year), using the Revised Seizure‐based Outcome Classification System (Duke).^19^ Adherence to medication was accessed using the Medication Adherence Rating Scale (MARS) that required the patient to answer 10 questions (≤5 is not adherent; ≥6 is adherent). Vaccination‐related variables included history of pre‐vaccination confirmed COVID‐19 infection, type of COVID‐19 vaccine (Cominarty®; ChAdOx1‐S®; CoronaVac®), duration of onset and outcome of AEFI. The vaccination details were clarified via the participants' national COVID‐19 mobile application–MySejahtera, which was developed by the Malaysian government to facilitate the management of COVID‐19 with individualized vaccination profiles. Local AEFI included injection site pain or tenderness, redness, swelling, and pruritus. Systemic AEFI included fever, fatigue, chills, nausea, vomiting, dysphagia, anorexia, constipation, diarrhea, abnormal skin, and mucosa at non‐injection site, new or worsened myalgia at non‐injection site, new or worsened arthralgia at non‐injection site, sore throat, acute allergy reaction, and use of antipyretic or analgesia. Non‐serious neurological AEFI included headache, sensory symptoms, weakness, and syncope. Serious neurological AEFI included Guillain‐Barré syndrome (GBS), acute transverse myelitis, seizure, acute palsy, or paralysis. The presentation of each AEFI was further categorized into none, requiring no hospital admission, or requiring hospital admission.

### 2.4 Statistical analyses

Data analysis of this study was performed using the Statistical Package for the Social Sciences (SPSS) version 28. Categorical data were expressed as frequencies and percentages, while continuous data were expressed in means and standard deviations. Fisher’s exact test (FE), Pearson’s chi‐square (PCS), and independent t‐test were used for univariate comparison. The associations between pre‐existing epilepsy and other variables were determined using the logistic regression analysis; the variables with *P* < 0.05 from the univariate analyses were entered into the multiple logistic regression analysis model. Odds ratios (ORs) and 95% confidence intervals (CIs) were estimated. A *P*‐value less than 0.05 (2‐sided) was considered significant.

## 3 RESULTS

### 3.1 Characteristics of PWE

The characteristics of the 120 PWE are shown in Table 1. The socio‐demographic characteristics along with epilepsy‐related and vaccines‐related variables are included.

**TABLE 1:** The characteristics of PWE

PWE (N = 120)	Characteristics	PWE, n (%)		
SOCIODEMOGRAPHIC CHARACTERISTICS				
Gender	Female	51 (42.5%)		
	Male	69 (57.5%)		
Race	Malay	57 (47.5%)		
	Chinese	47 (39.2%)		
	Indian	15 (12.5%)		
	Others	1 (0.8%)		
Age, years (mean ± standard deviation)		38.23 ± 16.771		
Body mass index, kg/m^2^ (mean ± standard deviation)		24.38 ± 6.100 (Normal)		
Categories of body mass index	Underweight	17 (14.2%)		
	Normal	37 (30.8%)		
	Overweight/Pre‐Obese	32 (26.7%)		
	Obese I	29 (24.2%)		
	Obese II	3 (2.5%)		
	Obese III	2 (1.7%)		
Education level	None	4 (3.3%)		
	Secondary school	54 (45.0%)		
	Pre‐university	17 (14.2%)		
	Undergraduate	26 (21.7%)		
	Post‐graduate	1 (0.8%)		
	Others	18 (15.0%)		
Marital status	Single	75 (62.5%)		
	Married	41 (34.2%)		
	Divorced	3 (2.5%)		
	Widowed	1 (0.8%)		
Employment status	Full Time Employment	46 (38.3%)		
	Part‐time Employment	5 (4.2%)		
	Self‐employed	3 (2.5%)		
	Student	8 (6.7%)		
	Retired	9 (7.5%)		
	Unemployed	49 (40.8%)		
Allergy history	Yes	15 (12.5%)		
	No	105 (87.5%)		
Other medical illnesses	Yes	80 (66.7%)	Congenital diseases	18 (15.0%)
			Metabolic syndromes	41 (34.2%)
			Mental disorders	19 (15.8%)
	No	40 (33.3%)		
History of surgery	Yes	43 (35.8%)		
	No	77 (64.2%)		
Alternative medication	Yes	11 (9.2%)		
	No	109 (90.8%)		
EPILEPSY‐RELATED VARIABLES				
Time interval from first onset of seizure to definite diagnosis of epilepsy, years (mean ± standard deviation)		1.23 ± 3.845 (range: 0 to 30 years; median: 0)		
Type of epilepsy	Focal	60 (50.0%)		
	Generalized	57 (47.5%)		
	Combined	1 (0.8%)		
	Unknown	2 (1.7%)		
Pre‐vaccination seizure frequency	Seizure free	38 (31.7%)		
	Lower seizure frequency	48 (40.0%)		
	Higher seizure frequency	34 (28.3%)		
Difference in seizure frequency between pre‐vaccination and post‐vaccination	Unchanged	101 (84.2%)		
	Increased	5 (4.2%)		
	Decreased	14 (11.7%)		
History of status epilepticus within 3 months before first dose of vaccination	Yes	12 (10.0%)		
	No	108 (90.0%)		
Number of current ASM	Monotherapy	51 (42.5%)	Carbamazepine	7 (5.8%)
			Lamotrigine	4 (3.3%)
			Levetiracetam	19 (15.8%)
			Phenytoin	6 (5.0%)
			Sodium valproate	14 (11.7%)
	Polytherapy	69 (57.5%)		
Adherence to medication	Adherent	95 (79.2%)		
	Non‐adherent	25 (20.8%)		
VACCINES‐RELATED VARIABLES				
History of pre‐vaccination confirmed COVID‐19 infection	Yes	5 (4.2%)		
	No	115 (95.8%)		
Type of COVID‐19 vaccine	Cominarty® (Pfizer‐BioNTech)	63 (52.5%)		
	ChAdOx1‐S® (Oxford‐AstraZeneca)	17 (14.2%)		
	CoronaVac® (Sinovac)	40 (33.3%)		

### 3.2 Frequency and duration of onset of AEFI following COVID‐19 vaccination

Overall, 75.8% of the participants experienced AEFI of COVID‐19 vaccination. The highest prevalence of AEFI was systemic AEFI (64.2%). The lowest prevalence of AEFI was serious neurological AEFI (3.3%). Local AEFI was the quickest to develop, with an average onset within a day requiring no hospital admission. Serious neurological AEFI was the slowest to present, with an average onset of 4 days with one index requiring hospital admission. The cumulative prevalence and duration of onset of AEFI of COVID‐19 vaccination are shown in Table 2.

**TABLE 2:** The frequency and duration of onset of AEFI following COVID‐19 vaccination

PWE (N = 120)		PWE, n (%)		PWE, n (%)
Local AEFI	None	58 (48.3%)		
	Time of onset to local AEFI, day (mean ± standard deviation)	0.00 ± 0.00	Local AEFI requiring no hospital admission	62 (51.7%)
			Local AEFI requiring hospital admission	‐
Systemic AEFI	None	43 (35.8%)		
	Time of onset to systemic AEFI, day (mean ± standard deviation)	0.02 ± 0.14	Systemic AEFI requiring no hospital admission	74 (61.7%)
			Systemic AEFI requiring hospital admission	3 (2.5%)
Non‐serious neurological AEFI	None	88 (73.3%)		
	Time of onset to non‐serious neurological AEFI, day (mean ± standard deviation)	1.05 ± 0.22	Non‐serious neurological AEFI requiring no hospital admission	31 (25.8%)
			Non‐serious neurological AEFI requiring hospital admission	1 (0.8%)
Serious neurological AEFI	None	116 (96.7%)		
	Time of onset to serious neurological AEFI, day (mean ± standard deviation)	4.00 ± 5.66	Serious neurological AEFI requiring no hospital admission	3 (2.5%)
			Serious neurological AEFI requiring hospital admission	1 (0.8%)
Overall prevalence of AEFI	None	29 (24.2%)		
	Time of onset to any AEFI, day (mean ± standard deviation)	0.14 ± 1.01	AEFI requiring no hospital admission	87 (72.5%)
			AEFI requiring hospital admission	4 (3.3%)

### 3.3 Clinical presentation of AEFI of COVID‐19 vaccination in PWE

The analytical clinical presentation of AEFI is shown in Table 3. People with epilepsy after the CoronaVac® vaccination reported the lowest prevalence of systemic AEFI, while PWE following the ChAdOx1‐S® vaccination had the highest prevalence of systemic AEFI (*P* = 0.03)**.** The most common local AEFI was injection site pain or tenderness. Regardless of the vaccination brands, it was equally high in prevalence for PWE to take antipyretics or analgesics to relieve their presenting AEFI. The two most common non‐serious neurological AEFI were headache and weakness. Furthermore,  those who were administered the CoronaVac® vaccine also reported the lowest prevalence of non‐serious neurological AEFI (*P* = 0.005). However, that particular patient required hospital admission for the presenting complaint.

**TABLE 3:** The clinical presentation of AEFI of COVID‐19 vaccines

PWE (N = 120)	Type of COVID‐19 vaccine	Cominarty® (Pfizer‐BioNTech), n = 63 PWE, n (%)	ChAdOx1‐S® (Oxford‐AstraZeneca), n = 17 PWE, n (%)	CoronaVac® (Sinovac), n = 40 PWE, n (%)	*P*
Difference in seizure frequency between pre‐vaccination and post‐vaccination (PCS)	Unchanged	53 (84.1%)	14 (82.4%)	34 (85.0%)	0.12
	Increased	1 (1.6%)	0 (0.0%)	4 (10.0%)	
	Decreased	9 (14.3%)	3 (17.6%)	2 (5.0%)	
Local AEFI (PCS)	None	26 (41.3%)	8 (47.1%)	24 (60.0%)	0.18
	Requiring no hospital admission	37 (58.7%)	9 (52.9%)	16 (40.0%)	
	Requiring hospital admission	0 (0.0%)	0 (0.0%)	0 (0.0%)	
Injection site pain/tenderness		34 (54.0%)	8 (47.1%)	14 (35.0%)	
Redness		4 (6.3%)	0 (0.0%)	3 (7.5%)	
Swelling		8 (12.7%)	3 (17.6%)	7 (17.5%)	
Pruritus		3 (4.8%)	0 (0.0%)	2 (5.0%)	
Systemic AEFI (PCS)	None	20 (31.7%)	2 (11.8%)	21 (52.5%)	0.03*
	Requiring no hospital admission	41 (65.1%)	15 (88.2%)	18 (45.0%)	
	Requiring hospital admission	2 (11.8%)	0 (0.0%)	1 (2.5%)	
Fever		17 (27.0%)	8 (47.1%)	6 (15.0%)	
Fatigue		26 (41.3%)	11 (64.7%)	11 (27.5%)	
Chills		11 (17.5%)	3 (17.6%)	1 (2.5%)	
Nausea		0 (0.0%)	4 (23.5%)	2 (5.0%)	
Vomiting		0 (0.0%)	1 (5.9%)	1 (2.5%)	
Dysphagia		0 (0.0%)	0 (0.0%)	0 (0.0%)	
Anorexia		1 (1.6%)	1 (5.9%)	3 (7.5%)	
Constipation		1 (1.6%)	0 (0.0%)	0 (0.0%)	
Diarrhea		2 (3.2%)	0 (0.0%)	0 (0.0%)	
Abnormal skin and mucosa (non‐injection site)		2 (3.2%)	0 (0.0%)	0 (0.0%)	
New/worsened myalgia (non‐injection site)		8 (12.7%)	3 (17.6%)	2 (5.0%)	
New/worsened arthralgia (non‐injection site)		6 (9.5%)	2 (11.8%)	0 (0.0%)	
Sore throat		1 (1.6%)	0 (0.0%)	0 (0.0%)	
Acute allergy reaction		1 (1.6%)	0 (0.0%)	1 (2.5%)	
Use of antipyretics/analgesia		29 (46.0%)	13 (76.5%)	12 (30.0%)	
Non‐serious neurological AEFI (PCS)	None	47 (74.6%)	7 (41.2%)	34 (85.0%)	0.005*
	Requiring no hospital admission	16 (25.4%)	10 (58.8%)	5 (12.5%)	
	Requiring hospital admission	0 (0.0%)	0 (0.0%)	1 (2.5%)	
Headache		8 (12.7%)	7 (41.2%)	5 (12.5%)	
Sensory symptoms		1 (1.6%)	0 (0.0%)	1 (2.5%)	
Weakness		9 (14.3%)	6 (35.3%)	3 (7.5%)	
Syncope		1 (1.6%)	1 (5.9%)	0 (0.0%)	
Serious neurological AEFI (PCS)	None	60 (95.2%)	17 (100.0%)	39 (97.5%)	0.31
	Requiring no hospital admission	3 (4.8%)	0 (0.0%)	0 (0.0%)	
	Requiring hospital admission	0 (0.0%)	0 (0.0%)	1 (2.5%)	
Guillain‐Barré syndrome		0 (0.0%)	0 (0.0%)	0 (0.0%)	
Acute transverse myelitis		0 (0.0%)	0 (0.0%)	0 (0.0%)	
Seizure		3 (4.8%)	0 (0.0%)	1 (2.5%)	
Acute palsy/paralysis		0 (0.0%)	0 (0.0%)	0 (0.0%)	

*Note:* Pearson’s chi‐square (PCS).

* *P‐*value less than 0.05 is considered as significant.

### 3.4 Association of characteristics of AEFI and COVID‐19 vaccination

Females had significantly more local or systemic AEFI than males (*P* = 0.02 and *P* = 0.007, respectively). The age group that developed systemic or non‐serious neurological AEFI was significantly younger than those who did not develop (*P* = 0.045 and *P* = 0.001, respectively). Most of the PWE, who developed either local or systemic AEFI, had significantly normal body mass index (*P* = 0.001 and *P* = 0.004, respectively). Race, education level, marital status, employment status, allergy history, other medical illnesses, history of surgery and alternative medications were not associated with any AEFI of COVID‐19 vaccination.

Serious neurological AEFI were significantly more frequent among PWE with a history of status epilepticus within 3 months before the first dose of COVID‐19 vaccination (*P* = 0.049). The time interval from the first onset of seizure to the diagnosis of epilepsy ranged from 0.25 to 1.29 years. The difference in seizure frequency between pre‐vaccination and post‐vaccination was insignificant. Otherwise, there was no significant association between other variables and AEFI of COVID‐19 vaccination. The detailed information and association between the characteristics and AEFI of COVID‐19 vaccination are summarized in Table 4.

**TABLE 4:** The characteristics of PWE associated with serious AEFI of COVID‐19 vaccination

PWE (N = 120)	AEFI	Local			Systemic			Non‐serious neurological			Serious neurological		
		None, n (%)	Yes, n (%)	*P*	None, n (%)	Yes, n (%)	*p*	None, n (%)	Yes, n (%)	*p*	None, n (%)	Yes, n (%)	*P*
SOCIODEMOGRAPHIC CHARACTERISTICS													
Gender (FE)	Female	18 (15.0%)	33 (27.5%)	0.02*	11 (9.2%)	40 (33.3%)	0.007*	33 (27.5%)	18 (15.0%)	0.09	49 (40.8%)	2 (1.7%)	1.00
	Male	40 (33.3%)	29 (24.2%)		32 (26.7%)	37 (30.8%)		55 (45.8%)	14 (11.7%)		67 (55.8%)	2 (1.7%)	
Race (PCS)	Malay	30 (25.0%)	27 (22.5%)	0.65	21 (17.5%)	36 (30.0%)	0.52	40 (33.3%)	17 (14.2%)	0.64	55 (45.8%)	2 (1.7%)	0.86
	Chinese	21 (17.5%)	26 (21.7%)		15 (12.5%)	32 (26.7%)		37 (30.8%)	10 (8.3%)		46 (38.3%)	1 (0.8%)	
	Indian	7 (5.8%)	8 (6.7%)		6 (5.0%)	9 (7.5%)		10 (8.3%)	5 (4.2%)		14 (11.7%)	1 (0.8%)	
	Others	0 (0.0%)	1 (0.8%)		1 (0.8%)	0 (0.0%)		1 (0.8%)	0 (0.0%)		1 (0.8%)	0 (0.0%)	
Age, years (mean ± standard deviation) (t)		39.86 ± 18.82	36.71 ± 14.56	0.31	42.33 ± 18.51	35.95 ± 15.37	0.05*	40.51 ± 18.44	31.97 ± 8.37	0.001*	38.38 ± 16.99	34.00 ± 8.49	0.61
Body mass index, kg/m^2^ (mean ± standard deviation) (t)		25.39 ± 7.16	23.47 ± 4.80	0.10	24.45 ± 6.53	24.34 ± 5.89	0.925	24.37 ± 6.21	24.43 ± 5.87	0.96	24.42 ± 6.16	23.25 ± 4.28	0.71
Categories of body mass index (PCS)	Underweight	12 (10.0%)	5 (4.2%)	0.001*	11 (9.2%)	6 (5.0%)	0.004*	15 (12.5%)	2 (1.7%)	0.46	17 (14.2%)	0 (0.0%)	0.94
	Normal	9 (7.5%)	28 (23.3%)		8 (6.7%)	29 (24.2%)		25 (20.8%)	12 (10.0%)		35 (29.2%)	2 (1.7%)	
	Overweight/Pre‐Obese	13 (10.8%)	19 (15.8%)		9 (7.5%)	23 (19.2%)		22 (18.3%)	10 (8.3%)		31 (25.8%)	1 (0.8%)	
	Obese I	21 (17.5%)	8 (6.7%)		12 (10.0%)	17 (14.2%)		22 (18.3%)	7 (5.8%)		28 (23.3%)	1 (0.8%)	
	Obese II	1 (0.8%)	2 (1.7%)		3 (2.5%)	0 (0.0%)		3 (2.5%)	0 (0.0%)		3 (2.5%)	0 (0.0%)	
	Obese III	2 (1.7%)	0 (0.0%)		0 (0.0%)	2 (1.7%)		1 (0.8%)	1 (0.8%)		2 (1.7%)	0 (0.0%)	
Education level (PCS)	None	3 (2.5%)	1 (0.8%)	0.66	2 (1.7%)	2 (1.7%)	0.33	4 (3.3%)	0 (0.0%)	0.46	4 (3.3%)	0 (0.0%)	0.98
	Secondary school	25 (20.8%)	29 (24.2%)		19 (15.8%)	35 (29.2%)		38 (31.6%)	16 (13.3%)		52 (43.8%)	2 (1.7%)	
	Pre‐university	7 (5.8%)	10 (8.3%)		8 (6.7%)	9 (7.5%)		12 (10.0%)	5 (4.2%)		17 (14.2%)	0 (0.0%)	
	Undergraduate	13 (10.8%)	13 (10.8%)		6 (5.0%)	20 (16.7%)		17 (14.2%)	9 (7.5%)		25 (20.8%)	1 (0.8%)	
	Post‐graduate	0 (0.0%)	1 (0.8%)		1 (0.8%)	0 (0.0%)		1 (0.8%)	0 (0.0%)		1 (0.8%)	0 (0.0%)	
	Others	10 (8.3%)	8 (6.7%)		7 (5.8%)	11 (9.2%)		16 (13.3%)	2 (1.7%)		17 (14.2%)	1 (0.8%)	
Marital status (PCS)	Single	36 (30.0%)	39 (32.5%)	0.72	29 (24.2%)	46 (38.3%)	0.42	55 (45.8%)	20 (16.7%)	0.93	73 (60.8%)	2 (1.7%)	0.91
	Married	21 (17.5%)	20 (16.7%)		12 (10.0%)	29 (24.2%)		30 (25.0%)	11 (9.2%)		39 (32.5%)	2 (1.7%)	
	Divorced	1 (0.8%)	2 (1.7%)		2 (1.7%)	1 (0.8%)		2 (1.7%)	1 (0.8%)		3 (2.5%)	0 (0.0%)	
	Widowed	0 (0.0%)	1 (0.8%)		0 (0.0%)	1 (0.8%)		1 (0.8%)	0 (0.0%)		1 (0.8%)	0 (0.0%)	
Employment status (PCS)	Full Time Employment	24 (20.0%)	22 (18.3%)	0.40	16 (13.3%)	30 (25.0%)	0.52	32 (26.7%)	14 (11.7%)	0.24	44 (36.7%)	2 (1.7%)	0.96
	Part‐time Employment	2 (1.7%)	3 (2.5%)		1 (0.8%)	4 (3.3%)		3 (2.5%)	2 (1.7%)		5 (4.2%)	0 (0.0%)	
	Self‐employed	1 (0.8%)	2 (1.7%)		1 (0.8%)	2 (1.7%)		2 (1.7%)	1 (0.8%)		3 (2.5%)	0 (0.0%)	
	Student	1 (0.8%)	7 (5.8%)		1 (0.8%)	7 (5.8%)		4 (3.3%)	4 (3.3%)		8 (6.7%)	0 (0.0%)	
	Retired	5 (4.2%)	4 (3.3%)		5 (4.2%)	4 (3.3%)		9 (7.5%)	0 (0.0%)		9 (7.5%)	0 (0.0%)	
	Unemployed	25 (1.3%)	24 (20.0%)		19 (15.8%)	30 (25.0%)		38 (31.7%)	11 (9.2%)		47 (39.2%)	2 (1.7%)	
Allergy history (FE)	Yes	4 (3.3%)	11 (9.2%)	0.10	3 (2.5%)	12 (10.0%)	0.25	9 (7.5%)	6 (5.0%)	0.22	15 (12.5%)	0 (0.0%)	1.00
	No	54 (45.0%)	51 (42.5%)		40 (33.3%)	65 (54.2%)		79 (65.8%)	26 (21.7%)		101 (84.2%)	4 (3.3%)	
Other medical illnesses (FE)	Yes	40 (33.3%)	30 (33.3%)	0.70	30 (25.0%)	50 (41.7%)	0.69	63 (52.5%)	17 (14.2%)	0.08	79 (65.8%)	1 (0.8%)	0.11
	No	18 (15.0%)	22 (18.3%)		13 (10.8%)	27 (22.5%)		25 (20.8%)	15 (12.5%)		37 (30.8%)	3 (2.5%)	
History of surgery (FE)	Yes	21 (17.5%)	22 (18.3%)	1.00	16 (13.3%)	27 (22.5%)	0.84	35 (29.2%)	8 (6.7%)	0.20	42 (35.0%)	1 (0.8%)	1.00
	No	37 (30.8%)	40 (33.3%)		27 (22.5%)	50 (41.7%)		53 (44.2%)	24 (20.0%)		74 (61.7%)	3 (2.5%)	
Alternative medications (FE)	Yes	5 (4.2%)	6 (5.0%)	1.00	4 (3.3%)	7 (5.8%)	1.00	7 (5.8%)	4 (3.3%)	0.48	10 (8.3%)	1 (0.8%)	0.32
	No	53 (44.2%)	56 (46.7%)		39 (32.5%)	70 (58.3%)		81 (67.5%)	28 (23.3%)		106 (88.3%)	3 (2.5%)	
EPILEPSY‐RELATED VARIABLES													
Time interval from first onset of seizure to definite diagnosis of epilepsy, years (mean ± standard deviation) (t)		1.19 ± 4.43	1.26 ± 3.24	0.92	1.12 ± 4.79	1.29 ± 3.24	0.82	1.20 ± 4.21	1.28 ± 2.63	0.92	1.26 ± 3.91	0.25 ± 0.50	0.61
Type of epilepsy (PCS)	Focal	31 (25.8%)	29 (24.2%)	0.71	25 (20.8%)	35 (29.2%)	0.47	46 (38.3%)	14 (11.7%)	0.67	56 (46.7%)	4 (3.3%)	0.25
	Generalized	26 (21.7%)	31 (25.8%)		17 (14.2%)	40 (33.3%)		40 (33.3%)	17 (14.2%)		57 (47.5%)	0 (0.0%)	
	Combined	0 (0.0%)	1 (0.8%)		0 (0.0%)	1 (0.8%)		1 (0.8%)	0 (0.0%)		1 (0.8%)	0 (0.0%)	
	Unknown	1 (0.8%)	1 (0.8%)		1 (0.8%)	1 (0.8%)		1 (0.8%)	1 (0.8%)		2 (1.7%)	0 (0.0%)	
Pre‐vaccination seizure frequency (PCS)	Seizure free	16 (13.3%)	22 (18.3%)	0.50	15 (12.5%)	23 (19.2%)	0.82	27 (22.5%)	11 (9.2%)	0.47	37 (30.8%)	1 (0.8%)	0.61
	Lower seizure frequency	23 (19.2%)	25 (20.8%)		17 (14.2%)	31 (25.8%)		38 (31.7%)	10 (8.3%)		47 (39.2%)	1 (0.8%)	
	Higher seizure frequency	19 (15.8%)	15 (12.5%)		11 (9.2%)	23 (19.2%)		23 (19.2%)	11 (9.2%)		32 (26.7%)	2 (1.7%)	
Difference in seizure frequency between pre‐vaccination and post‐vaccination (PCS)	Unchanged	51 (42.5%)	50 (41.7%)	0.37	37 (30.8%)	64 (53.3%)	0.28	76 (63.3%)	25 (20.8%)	0.34	98 (81.7%)	3 (2.5%)	0.66
	Increased	1 (0.8%)	4 (3.3%)		3 (2.5%)	2 (1.7%)		4 (3.3%)	1 (0.8%)		5 (4.2%)	0 (0.0%)	
	Decreased	6 (5.0%)	8 (6.7%)		3 (2.5%)	11 (9.2%)		8 (6.7%)	6 (5.0%)		13 (10.8%)	1 (0.8%)	
History of status epilepticus within 3 months before first dose of vaccination (FE)	Yes	6 (5.0%)	6 (5.0%)	1.00	3 (2.5%)	9 (2.5%)	0.53	10 (8.3%)	2 (1.7%)	0.51	10 (8.3%)	2 (1.7%)	0.05*
	No	52 (43.3%)	56 (46.7%)		40 (33.3%)	68 (56.7%)		78 (65.0%)	30 (25.0%)		106 (88.3%)	2 (1.7%)	
Number of current ASM (FE)	Monotherapy	27 (22.5%)	24 (20.0%)	0.46	22 (18.3%)	29 (24.2%)	0.18	38 (31.7%)	13 (10.8%)	0.84	51 (42.5%)	0 (0.0%)	0.14
	Polytherapy	31 (25.8%)	38 (31.7%)		21 (17.5%)	48 (40.0%)		50 (41.7%)	19 (15.8%)		65 (54.2%)	4 (3.3%)	
Adherence to medication (FE)	Adherent	47 (39.2%)	48 (40.0%)	0.66	36 (30.0%)	59 (49.2%)	0.48	70 (58.3%)	25 (20.8%)	1.00	91 (75.8%)	4 (3.3%)	0.58
	Non‐adherent	11 (9.2%)	14 (11.7%)		7 (5.8%)	18 (15.0%)		18 (15.0%)	7 (5.8%)		25 (20.8%)	0 (0.0%)	
VACCINES‐RELATED VARIABLES													
History of pre‐vaccination confirmed COVID‐19 infection (FE)	Yes	2 (1.7%)	3 (2.5%)	1.00	2 (1.7%)	3 (2.5%)	1.00	3 (2.5%)	2 (1.7%)	0.61	4 (3.3%)	1 (0.8%)	0.16
	No	56 (46.7%)	59 (49.2%)		41 (34.2%)	74 (61.7%)		85 (70.8%)	30 (25.0%)		112 (93.3%)	3 (2.5%)	

*Note:* Pearson’s chi‐square (PCS), Fisher’s exact test (FE), and independent *t*‐test (t).

* *P‐*value less than 0.05 (2‐sided) is considered as significant.

All the characteristics and variables with a *P*‐value <0.05 were further analyzed with each category of the AEFI in a multiple logistic regression analysis model. The findings of the multivariate analyses indicated that there was no significant association between all characteristics of PWE with non‐serious neurological and serious neurological AEFI. The logistic regression multivariate analysis of the significant associations between characteristics of PWE and AEFI are shown in Table 5. Gender, age, history of status epilepticus within 3 months before the first dose of vaccination, and brand of COVID‐19 vaccine were not found to be associated with local or systemic AEFI. However, body mass index was found to be a significant factor associated with local or systemic AEFI. Only normal body mass index showed statistically significant association with systemic AEFI, compared to underweight PWE.

**TABLE 5:** The multiple logistics regression results of significant associations between characteristics of PWE and AEFI of COVID‐19 vaccination

	AEFI	Local^a^		Systemic^b^	
PWE (N = 120)	Characteristics	Odds ratio (95% confidence interval)	*P*	Odds ratio (95% confidence interval)	*P*
Gender	Male^c^				
	Female	1.86 (0.75–4.71)	0.18	2.09 (0.90–4.86)	0.09
Age		1.00 (0.97–1.03)	0.90	1.00 (0.97–1.02)	0.75
Categories of Body Mass Index	Underweight^c^				
	Normal	15.09 (1.70–134.28)	0.02*	9.20 (1.72–49.16)	0.01*
	Overweight/Pre‐Obese	10.66 (1.22–92.86)	0.03*	5.11 (1.00–27.06)	0.06
	Obese I	2.11 (0.20–22.69)	0.54	5.38 (0.98–29.38)	0.05
	Obese II	24.30 (1.00–591.17)	0.05*	‐	1.00
	Obese III	‐	1.00	‐	1.00
History of status epilepticus within 3 months before first dose of vaccination	Yes^c^				
	No	0.402 (0.07–2.26)	0.30	0.94 (0.25–3.51)	0.92
Type of COVID‐19 vaccine	Cominarty® (Pfizer‐BioNTech)^c^				
	ChAdOx1‐S® (Oxford‐AstraZeneca)	0.52 (0.18–1.46)	0.21	0.50 (0.198–1.241)	0.13
	CoronaVac® (Sinovac)	0.36 (0.09–1.49)	0.16	0.32 (0.088–1.190)	0.09

^a^ Naglerke R^2^ = 0.29.

^b^ Naglerke R^2^ = 0.24.

^c^ Reference.

* *P‐*value less than 0.05 is considered as significant.

### 3.5 Characteristics of PWE associated with serious AEFI

Out of the 120 PWE, only four patients developed serious AEFI following COVID‐19 vaccination. All patients were younger than 45 years of age. Three patients were vaccinated with Cominarty® and one patient was vaccinated with CoronaVac®. The serious neurological AEFI experienced was seizure, and it occurred within 1‐8 days after vaccination. It is important to emphasize that all patients had at least one systemic or non‐serious neurological AEFI. The detailed characteristics and variables with a *P* value of <0.05 were tabulated in Table 6.

**TABLE 6:** The characteristics of PWE associated with serious AEFI of COVID‐19 vaccination

Index	Gender	Age	Category of body mass index	Difference in seizure frequency between pre‐vaccination and post‐vaccination	History of status epilepticus within 3 months before first dose of vaccination	Type of COVID‐19 vaccine	Time of onset to serious neurological AEFI, day	Serious neurological AEFI experienced	Presentation of serious neurological AEFI – seizure
#10	Male	40	Obese I	Unchanged	No	CoronaVac® (Sinovac)	8	Seizure	Requiring hospital admission.
#28	Male	30	Normal	Unchanged	Yes	Cominarty® (Pfizer‐BioNTech)	0	Seizure	Requiring no hospital admission.
#102	Female	42	Overweight / Pre‐Obese	Decreased	No	Cominarty® (Pfizer‐BioNTech)	1	Seizure	Requiring no hospital admission.
#120	Female	24	Normal	Unchanged	Yes	Cominarty® (Pfizer‐BioNTech)	1	Seizure	Requiring no hospital admission.

## 4 DISCUSSION

This study revealed significant associations between gender, age, BMI, and AEFI. More females reported local and systemic AEFI (64.7%, 78.4%) than males (42.0%, 53.6%). This finding is consistent with other papers with reasons postulated by Miguel and colleagues. It was thought to be due to sexual dimorphism in human immune response to vaccination where by female has a higher immune reactogenicity, hence more AEFI.^13^, ^14^, ^19^, ^20^, ^21^, ^22^

The average age range that was statistically significant with systemic and non‐serious neurological AEFI was less than 40 years old. Similarly, the vaccine clinical trials by Pfizer, CanSino and Johnson & Johnson, reported that younger participants (<55 years old) have a higher prevalence of AEFI.^23^, ^24^, ^25^ The age range prevalence distribution was supported by Polack and colleagues, who found a higher prevalence of systemic events among younger COVID‐19 vaccine recipients.^23^ Interestingly, our study reported that PWE with normal BMI had a higher risk of developing both local and systemic AEFI compared to those underweight and obese PWE. Iguacel and colleagues reported similar findings, in which there was a higher prevalence of local and systemic AEFI among COVID‐19 vaccination recipients of underweight and normal BMI. A lower antibody titre in recipients with central obesity was postulated to cause lesser AEFI among the overweight and obese COVID‐19 recipients albeit another study found no correlation between antibody titre and severity of symptoms.^14^, ^20^, ^26^, ^27^, ^28^, ^29^

In our cohort, two out of twelve PWE with a history of status epilepticus within 3 months prior to vaccination developed serious neurological AEFI. Status epilepticus is an imperative indicator of the underlying disease severity and seizure control.^26^ However, these two participants already had high seizure frequency prior to vaccination, and their serious neurological AEFI might not be caused by vaccination.

For participants with a history of pre‐vaccination COVID‐19 infection, the frequency of AEFI, especially of systemic AEFI was expected to be increased as suggested by Krammer and colleagues.^30^ Thomas and colleagues also reported that 12% of individuals, who had a history of pre‐vaccination COVID‐19 infection, reported fever compared to 3% of those without previous infection after the administration of the first dose of Cominarty®.^31^ Most AEFI were proposed to be the effect of pyrogenic cytokines causing more systemic reactions in an individual having had a history of COVID‐19 infection rendering an existing immunity.^32^ Vaccinated individuals with a history of COVID‐19 infection were shown to have 10‐45 times higher antibody titre, which may explain the increased AEFI.^30^ However, our finding suggested that pre‐vaccination confirmed COVID‐19 infection was not significantly correlated with the prevalence of any AEFI. A larger sample size with history of pre‐vaccination COVID‐19 infection is required for a better analysis.

When the prevalence of AEFI was compared between monotherapy and polytherapy of ASM, our findings were similar to a study by Özdemir and colleagues, which revealed no significant association with AEFI.^33^ Although statistically less significant (*P* = 0.46, *P* = 0.18, *P* = 0.84), the prevalence of AEFI was higher among participants taking polytherapy in all categories (local, systemic, and non‐serious neurological), while four participants who developed serious neurological AEFI were all on polytherapy (*P* = 0.14). Another paper by Von Wrede and colleagues also reported the similar findings.^20^ Polytherapy was not found to be correlated with AEFI, but a lower number of ASM can predict AEFI correctly at 83.3%. This was contrary to the findings reported by Massoud and colleagues, which stated that there is a higher prevalence of AEFI among patients receiving polytherapy.^14^ A larger sample size is required to investigate the association between the number of ASM and AEFI. The data collected on the number of ASM was based on the prescription at the time of interview, without considering the previous regimes of ASM. It was postulated that COVID‐19 vaccines might have interactions with ASM. COVID‐19 vaccines were able to induce a strong B‐cell response in producing antibodies and stimulating T‐cell response to produce interferon gamma, which subsequently resulted in downregulation of CYP1A2 and CYP3A4.^34^ This might lead to potential toxicity of ASM, particularly carbamazepine as reported by Robertson.^35^ Despite the absence of ASM toxicity in this cohort, healthcare providers should be vigilant in observing potential ASM toxicity.

This cross‐sectional study showed that the three COVID‐19 vaccines administered in Malaysia are safe for PWE. Our findings were supported by real‐world data. The most common local AEFI in our cohort was injection site pain or tenderness, similar to the findings reported in the phase 3 clinical trial of Cominarty®, and a phase 3 Indonesian study of CoronaVac®.^31^, ^36^ Unequivocally, the phase 3 safety and efficacy of ChAdOx1‐S® reported general pain as the most common local AEFI, followed by injection site pain.^37^ Apart from the usage of antipyretics or analgesia, the next most common systemic AEFI was fatigue, which was also supported by the phase 3 clinical trial of Cominarty®.^31^ The prevalence of systemic AEFI (64.2%) in our current study was lower than the clinical trial of ChAdOx1‐S® (71.6%), which might be due to lesser reactogenicity among PWE.^37^ The prevalence of systemic and non‐serious neurological AEFI was the highest among participants, who were vaccinated with ChAdOx1‐S®. The reported prevalence of AEFI post‐ChAdOx1‐S® vaccination was also the highest among the three vaccines.^31^, ^36^, ^37^

The present study did not include etiology of epilepsy as a variable as the aetiologies of several participants were yet to be identified. Only the type of epilepsy was included in the study and there was no significant association with any AEFI. However, the prevalence of AEFI post‐COVID‐19 vaccination was noted to be low among patients with specific epilepsy aetiologies. Hood and colleagues suggested that COVID‐19 vaccines were safe to be administered among patients with Dravet syndrome. In a separate study, Lu and colleagues reported that patients with tuberous sclerosis complex tolerated inactivated COVID‐19 vaccines well with only 25% of them developing mild AEFI.^38^, ^39^

There were only 5 (4.2%) PWE, who had an increase in frequency of seizures following vaccination in our study. Only 4 (3.3%) PWE developed serious neurological AEFI with only one of them requiring hospitalization. Other studies reported similar findings. Massoud and colleagues reported a low prevalence of seizure worsening among PWE after COVID‐19 vaccination.^14^ Several studies have also noted that there was no increase in seizure frequency among PWE, who were vaccinated against COVID‐19.^33^, ^40^ The ILAE recommends PWE to be vaccinated against COVID‐19.^9^ This study showed that the vaccines were well tolerated among the PWE with only a small increase in their seizure frequency.

## 5 LIMITATIONS

There are some limitations in our study. Our study did not include etiologies of epilepsy. The effects of different ASM on AEFI were not studied. This is a single center study with a relatively smaller sample size, which might not allow us to generalize the findings. ^31^

## 6 CONCLUSION

COVID‐19 vaccines are safe for PWE. Adverse events following COVID‐19 vaccination are similar to those of the general population in terms of the prevalence, symptoms experienced, and factors related to a higher prevalence of AEFI such as age and gender. Clinicians should encourage their patients with epilepsy to take the COVID‐19 vaccines.

## CONFLICTS OF INTEREST

The authors have no competing interests and relevant financial interest to disclose. The authors confirm that they have read the Journal’s position on issues involved in ethical publication and affirm that this report is consistent with those guidelines.

## Supporting information


Figure S1
