# Supplementary figures and images for: Safety and adverse events following COVID‐19 vaccination among people with epilepsy: A cross‐sectional study
Source: Epilepsia Open. 2022 Nov 21;8(1):60–76. doi: 10.1002/epi4.12658 (PMC9874900; doi:10.1002/epi4.12658)

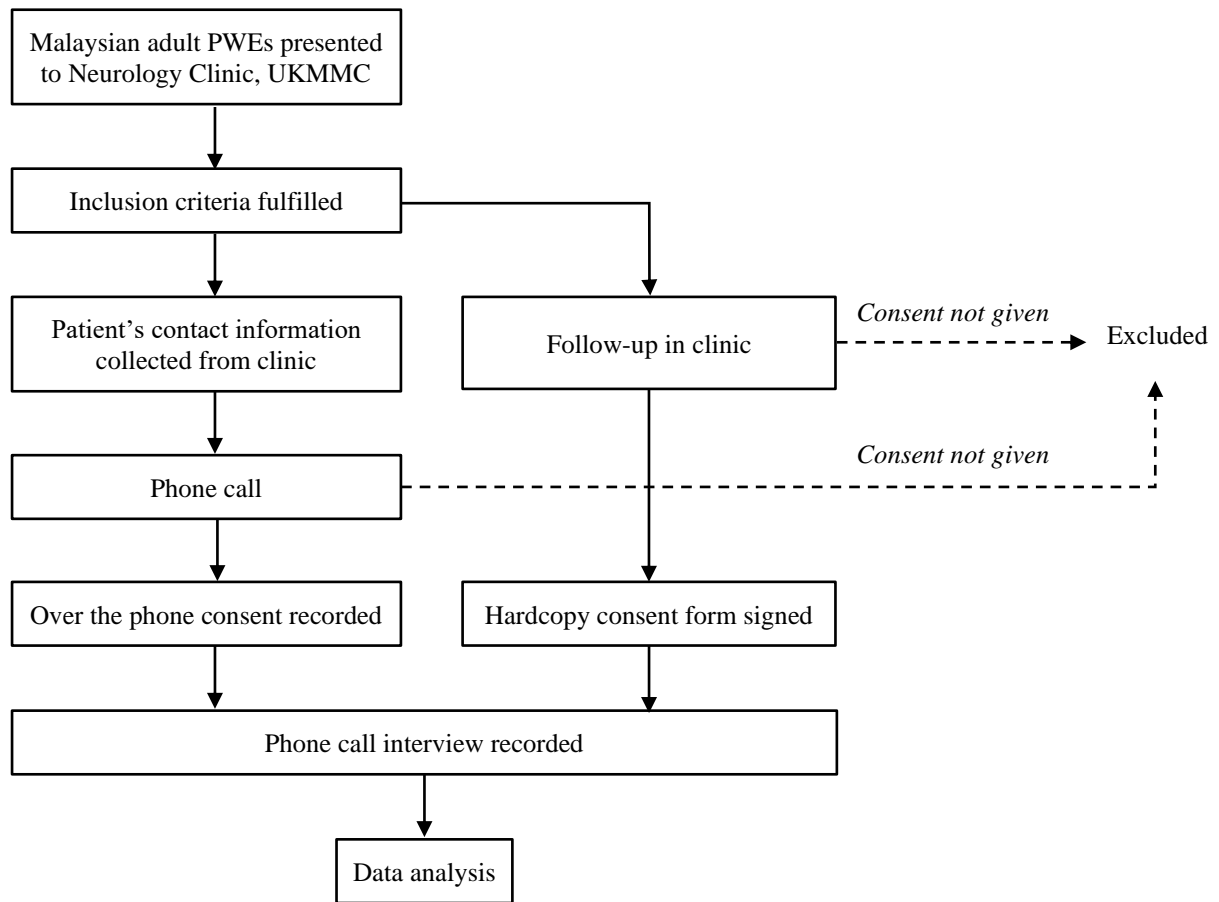

**Figure 1** Study flow chart.

Supplement: Supplementary file 1 — Figure S1 [file EPI4-8-60-s001.pdf]
